# Supplementary material for: Visible Light-Driven p-Type Semiconductor Gas Sensors Based on CaFe2O4 Nanoparticles
Source: Sensors (Basel). 2020 Feb 5;20(3):850. doi: 10.3390/s20030850 (PMC7038740; doi:10.3390/s20030850)
Supplement: Supplementary file 1 [file sensors-20-00850-s001.pdf]

# Supplementary Information

# Visible Light-Driven *p*-Type Semiconductor Gas Sensors Based on $CaFe_2O_4$ Nanoparticles †


**Qomaruddin [1,2,3,*], Olga Casals [1], Andris Šutka [4], Tony Granz [2], Andreas Waag [2], Hutomo Suryo Wasisto [2], Joan Daniel Prades [1,*] and Cristian Fàbrega [1,*]**

1 MIND-IN2UB, Department of Electronic and Biomedical Engineering, Universitat de Barcelona, Barcelona 08028, Spain; ocasals@el.ub.edu (O.C.)

2 Institute of Semiconductor Technology (IHT) and Laboratory for Emerging Nanometrology (LENA), Technische Universität Braunschweig, Braunschweig 38106, Germany; t.granz@tu-braunschweig.de (T.G.); a.waag@tu-braunschweig.de (A.W.); h.wasisto@tu-braunschweig.de (H.S.W.)

3 Research Center for Physics, Indonesian Institute of Sciences (LIPI), Tangerang Selatan 15314, Indonesia

4 Research Laboratory of Functional Materials Technologies, Faculty of Materials Science and Applied Chemistry, Riga Technical University, Riga 1048, Latvia; andris.sutka@rtu.lv

* Correspondence: qomaruddin@el.ub.edu (Q.); cfabrega@el.ub.edu (C.F.); dprades@el.ub.edu (J.D.P.)


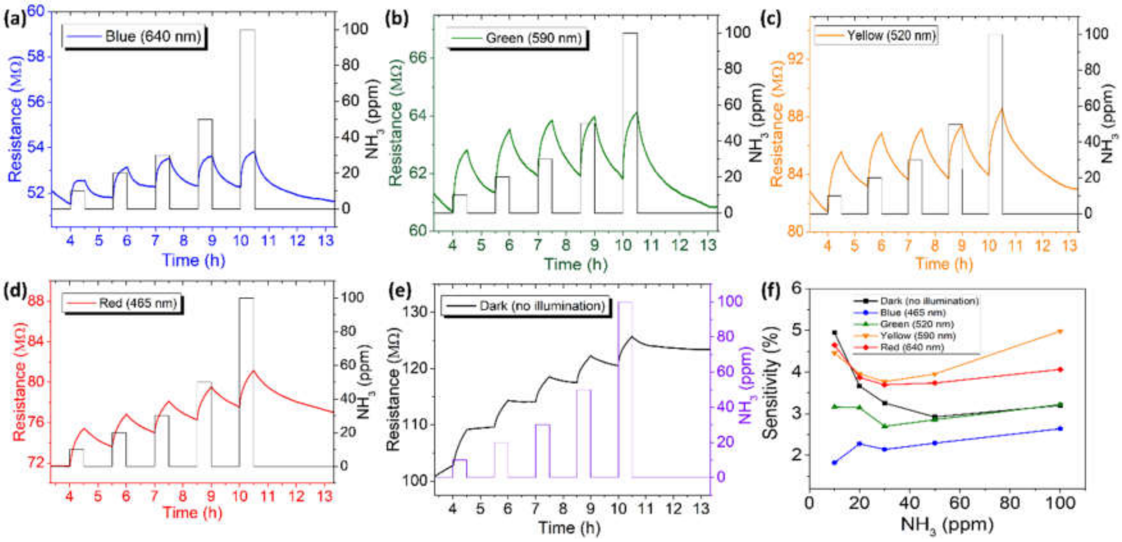


**Figure S1.** Dynamic responses of $CaFe_2O_4$ towards $NH_3$ reducing gas in varied vapor concentrations (i.e., 10, 20, 30, 50, and 100 ppm) under light activation from (**a**) blue (465 nm), (**b**) green (520 nm), (**c**) yellow (590 nm), (**d**) red (640 nm) LEDs and (**e**) in dark condition (without illumination). (**f**) Comparison of the sensor sensitivity under visible light exposures and dark condition for $NH_3$ sensing.

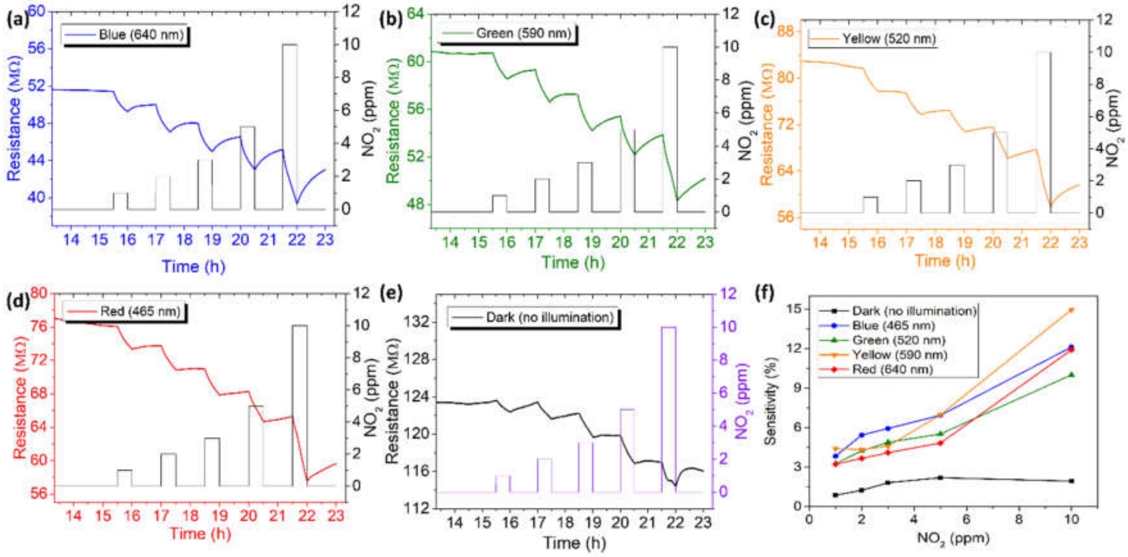


**Figure S2.** Dynamic responses of $CaFe_2O_4$ towards $NO_2$ gas in varied vapor concentrations (i.e. 1, 2, 3, 5, and 10 ppm) under light activation from (**a**) blue (465 nm), (**b**) green (520 nm), (**c**) yellow (590 nm), (**d**) red (640 nm) LEDs and (**e**) in dark condition (without illumination). (**f**) Comparison of the sensor sensitivity under visible light exposures and dark condition for $NO_2$ sensing.
